# Variations in the investigation of colorectal cancer-related symptoms in Australian primary care: a retrospective cohort study

**DOI:** 10.3399/BJGP.2025.0619

**Published:** 2026-02-24

**Authors:** Shaoke Lei, Brent Venning, Alison Pearce, Jon Emery

**Affiliations:** 1 Department of General Practice and Primary Care, University of Melbourne, Melbourne, Australia; 2 Collaborative Centre for Genomic Cancer Medicine, University of Melbourne, Melbourne, Australia; 3 Daffodil Centre, University of Sydney, a joint venture with Cancer Council NSW, Sydney, Australia; 4 Sydney School of Public Health, University of Sydney, Sydney, Australia; 5 Population and Global Health, Lee Kong Chian School of Medicine, Nanyang Technological University, Singapore, Singapore

**Keywords:** clinical decision-making, colorectal cancer, diagnostic techniques and procedures, early detection of cancer, general practice, healthcare disparities

## Abstract

**Background:**

Colorectal cancer-related symptoms are commonly seen in primary care. Understanding variations in symptom management is crucial for timely care.

**Aim:**

To examine diagnostic testing patterns for gastrointestinal symptoms associated with colorectal cancer in primary care.

**Design and setting:**

Retrospective cohort study using linked primary care data from patients aged ≥40 years presenting with colorectal cancer-related symptoms between 2008 and 2022.

**Method:**

The proportion of patients receiving diagnostic actions was assessed, including medications, pathology, imaging, colonoscopy, or specialist referral. Differences in testing by socioeconomic status and subsequent colorectal cancer diagnosis were examined.

**Results:**

Among 70 107 patients, 378 (0.5%) were diagnosed with colorectal cancer within 12 months. Abdominal pain (32%; *n* = 28 455/90 059) and diarrhoea (21%; *n* = 19 071/90 059) were the most frequent presentations. No investigation or treatment occurred in 20% (change in bowel habit; *n* = 226/1106) to 50% (constipation; *n* = 6028/12 128; and diarrhoea; *n* = 7069/14 122) of patients. Medication-only management was highest for diarrhoea (11%; *n* = 1547/14 122) and constipation (23%; *n* = 2847/12 128). Blood or imaging tests were most frequent for abdominal pain (36%; *n* = 7572/20 956) and abdominal mass (41%; *n* = 294/718). Colonoscopy or specialist referral was highest for rectal bleeding (67%; *n* = 4536/6772) and change in bowel habit (60%; *n* = 662/1106). Investigation likelihood increased with multiple symptoms or repeat visits (odds ratio [OR] 3.40, 95% confidence interval [CI] = 3.16 to 3.65, *P*<0.001). Patients aged ≥80 years were significantly less likely to be investigated or referred (OR 0.53, 95% CI = 0.50 to 0.55, *P*<0.001), and those from more advantaged areas were more likely to be investigated (OR 1.45, 95% CI = 1.38 to 1.53, *P*<0.001).

**Conclusion:**

Substantial variation in investigating colorectal cancer-related symptoms may contribute to inequities in diagnostic outcomes.

## How this fits in

Previous studies have focused on patients with confirmed colorectal cancer diagnoses, providing limited guidance for managing patients with undifferentiated symptomatic presentations. Even ‘red flag’ symptoms like rectal bleeding and anaemia had positive predictive values <2% for colorectal cancer in this real-world cohort. Investigation rates varied substantially by age and socioeconomic status, with patients aged ≥80 years and from disadvantaged areas receiving fewer investigations despite similar symptom presentations. GPs need systematic, evidence-based approaches to investigate lower gastrointestinal symptoms that balance cancer detection with avoiding overinvestigation.

## Introduction

In Australia, colorectal cancer is the fourth most commonly diagnosed cancer and second leading cause of cancer death, with over 15 000 new diagnoses and 5000 deaths annually.^1^ Although incidence has declined among those aged >50 years, rates are rising in younger populations.^2^ Five-year survival exceeds 98% for stage one disease but falls below 15% for stage four.^1^

Primary care plays a central role in colorectal cancer diagnosis. Australia’s National Bowel Cancer Screening Program (NBCSP) provides free biennial immunochemical faecal occult blood tests (iFOBT) to individuals aged 50–74 years, but participation remains low at 41.7%.^3^ Most bowel cancers are detected when patients present symptomatically to general practice.

Timely diagnosis improves outcomes, but most colorectal cancer-related symptoms have low predictive value, complicating GP decision making.^4–6^ Although Australian patients presenting with ‘alarm’ symptoms, such as rectal bleeding, experience shorter diagnostic intervals, many patients with colorectal cancer present with other symptoms, complicating timely recognition and investigation.^7,8^

The existing literature focuses on retrospective analyses of patients with diagnosed colorectal cancer,^9^ with limited evidence on the investigation of patients with undifferentiated symptomatic presentations. This study examined diagnostic testing and management patterns for colorectal cancer-related symptoms in primary care and identified patient characteristics contributing to variations in diagnostic assessment.

## Method

### Data sources

The datasets used in the study include the general practice electronic health record PATRON,^10^ the Victorian Admitted Episodes Dataset (VAED), and the Victorian Cancer Registry (VCR).^9,11^ PATRON contains GP encounter information for approximately 1.5 million de-identified patients from 130 Victorian general practices from 2008 to 2022.^10^ VAED contains data on admitted patient episodes to public and private hospitals in Victoria from 2008 to 2022.^11^ Records for patients diagnosed with cancer in Victoria during the same period were sourced from the VCR.^9^ The Centre for Victorian Data Linkage (CVDL) conducted the data linkage to link patient records from these datasets.^12^

### Symptoms associated with colorectal cancer

The colorectal cancer-related symptoms examined in this study were identified based on the Australian optimal care pathway.^13^ Symptom data were sourced from the ‘reason for encounter’ field using a comprehensive list of synonyms (see Supplementary Information S1 and Supplementary Box S1), developed with clinicians (see Supplementary Figure S1).

### Patient cohort

**Figure 1.**
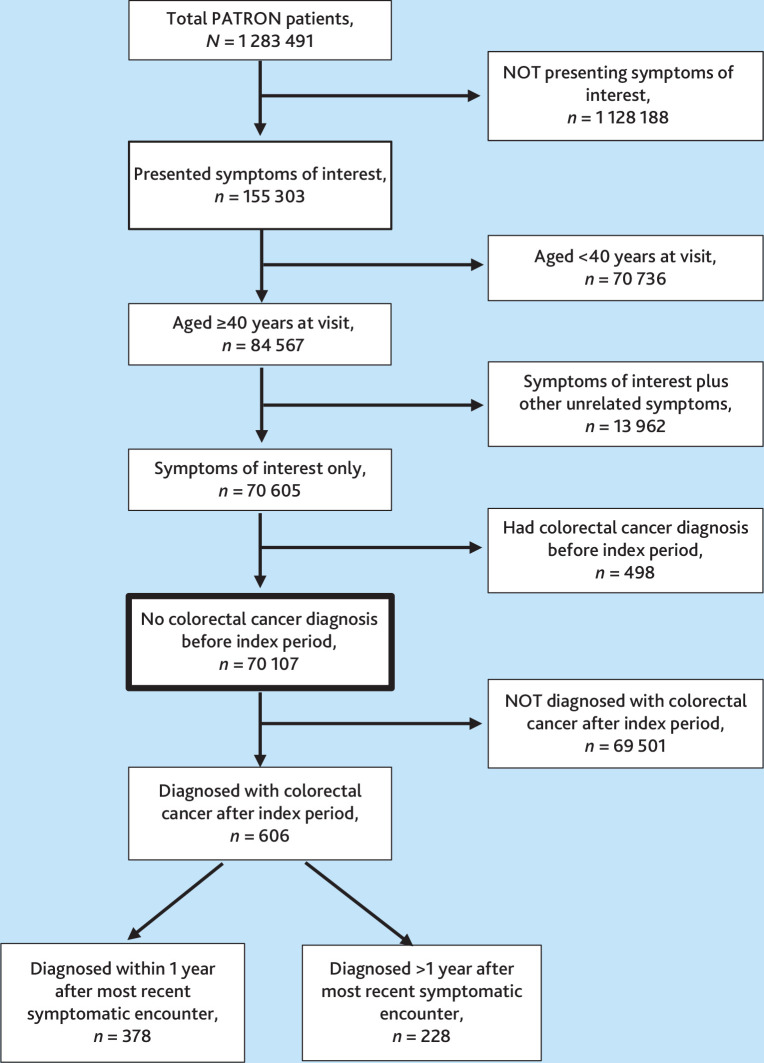
Flowchart outlining the steps involved in selecting the final patient cohort for the study.

Figure 1 outlines the cohort identification methodology. The most recent GP encounter for individuals aged ≥40 years in which any symptom of interest was recorded was selected, ensuring a comprehensive review of prior symptom history within a defined timeframe. The preceding 12 months were then examined, referred to as the index period, to identify any additional relevant symptoms. If an earlier symptom was found, that date marked the beginning of the index period, and patterns of investigation were assessed for the following 12 months.

Each patient was included only once in the cohort, based on their index period, while symptom combinations were recorded to reflect clinical complexity rather than assigning patients to separate symptom-specific cohorts. When multiple symptoms occurred within the index period, it was assumed they were related to the same illness. For example, if abdominal pain and constipation were recorded at the same encounter, they were treated as a combined presentation rather than counted separately. Encounters that contained multiple unrelated issues within the same consultation were excluded as otherwise this made it difficult to determine which investigations were associated with which presenting problem.

Of 84 567 patients initially identified, 70 107 remained after excluding patients with multiple unrelated symptoms and prior colorectal cancer diagnoses. The cohort was linked to VCR (International Classification of Diseases-10-AM codes C18–C20) to identify cancer diagnoses within 12 months of symptomatic presentation; those with patient numbers <5 were excluded as per CVDL requirements.

Demographic data were obtained from the PATRON database at the time of the symptomatic encounter. They included patient age, sex, remoteness classification,^14^ Index of Relative Socioeconomic Disadvantage,^15^ and smoking status.

### Investigations

A targeted list of relevant investigations was compiled to include diagnostic tests commonly associated with colorectal cancer, as well as those pertinent to other potential differential diagnoses corresponding to the presenting symptoms. The analysis was restricted to investigations conducted within primary care at the time of the symptom-related encounter. Tests ordered outside of this timeframe, either before or after the index consultation, were excluded because it was not possible to confirm their association with the presenting complaint. The specific investigations extracted from primary care data are detailed in Supplementary Box S2. As referral information is recorded as free text in PATRON, relevant referral letters were captured from the same symptomatic encounter. Whether patients underwent colonoscopy at a Victorian hospital within 12 months after their general practice visit was determined using the VAED.

### Medications used as a ‘test of treatment’

The term ‘test of treatment’ refers to the clinical approach of assessing a patient’s response to a specific therapy to support or rule out potential diagnoses.^16^ A targeted list of medications was compiled based on the Australian Medicines Handbook, focusing specifically on those agents that may have been used as tests of treatment for lower gastrointestinal symptoms associated with colorectal cancer (see Supplementary Box S3).^10^

### Statistical analysis

The frequency of symptom presentations in general practice was calculated, and patient characteristics were described based on whether they underwent further investigation or not. Categorical data were compared using a χ^2^ two-sample test. To examine factors associated with diagnostic testing or referral, a multiple logistic regression model was applied, with variable selection performed through backward elimination based on the Akaike information criterion. To examine practice-level variation in investigation patterns, a multilevel logistic regression model with random intercepts for practices was fitted. The model included all patient-level covariates identified in the primary logistic regression analysis. The odds ratio (OR) was calculated covering the middle 95% of practices to quantify the extent of practice-level variation independent of patient characteristics. The proportion of patients diagnosed with colorectal cancer within 12 months of presentation was described by calculating the ratio of total patients with cancer per symptom to the total number of patients presenting with that symptom, as well as the ratio of total patients with cancer to the entire cohort size. The time to diagnosis was estimated using median and interquartile ranges for the time to diagnosis in days from the first symptomatic encounter. All datasets underwent cleaning, management, and analysis using R (version 4.3.1), which were conducted securely on the online platform of the Victorian data Access Linkage Trust.

## Results

### Variation in investigations or referral

**Figure 2.**
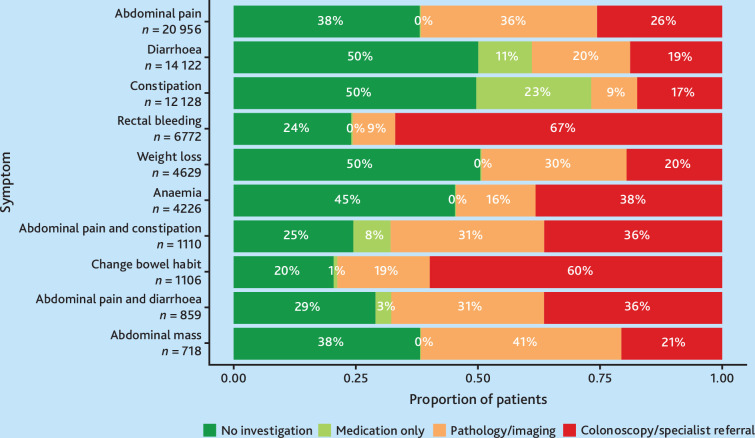
Proportion of patients with colorectal cancer-related symptoms managed by either no investigation, medication only, pathology/imaging, or colonoscopy/specialist referral. Symptoms are listed in order of the 10 most common colorectal cancer symptoms across patients.

Abdominal pain (32%; *n* = 28 455/90 059) and diarrhoea (21%; *n* = 19 071/90 059) were the most frequent presentations (see Supplementary Figure S2). Figure 2 illustrates investigation and management patterns. No investigation occurred in 20% (change in bowel habit; *n* = 226/1106) to 50% (constipation; *n* = 6028/12 128; and diarrhoea; *n* = 7069/14 122) of patients. Medication-only management (‘test of treatment’) predominated for diarrhoea (11%; *n* = 1547/14 122) and constipation (23%; *n* = 2847/12 128), whereas blood or imaging tests were common for abdominal pain (36%; *n* = 7572/20 956) and abdominal mass (41%; *n* = 294/718). Colonoscopy or specialist referral was highest for rectal bleeding (67%; *n* = 4536/6772) and change in bowel habit (60%; *n* = 662/1106).

**Figure 3.**
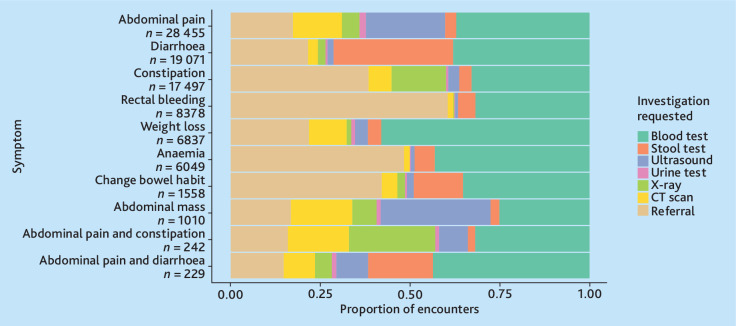
Variation of investigations ordered for colorectal cancer-related symptoms. Symptoms are listed in order of the 10 most common colorectal cancer symptoms by reason for encounter. CT = computed tomography.

Figure 3 presents diagnostic investigation variations across the 10 most common symptoms. Blood tests predominated, particularly for weight loss (58%), abdominal pain (37%), and diarrhoea (38%). Stool tests were common for diarrhoea (33%) and change in bowel habit (17%). Out of 4206 stool tests, 1197 (28%) tests included an iFOBT (see Supplementary Figure S3). Imaging was frequent for abdominal mass (ultrasound 31%, computed tomography [CT] scan 17%, and X-ray 7%) and abdominal pain and constipation (ultrasound 8%, CT scan 17%, and X-ray 24%) (Figure 3). Specialist referrals occurred in 60% for rectal bleeding and 48% for anaemia, versus 16% for abdominal pain and constipation. Urine tests were infrequently used across all symptom groups (0.2%–1.8%).

**Figure 4.**
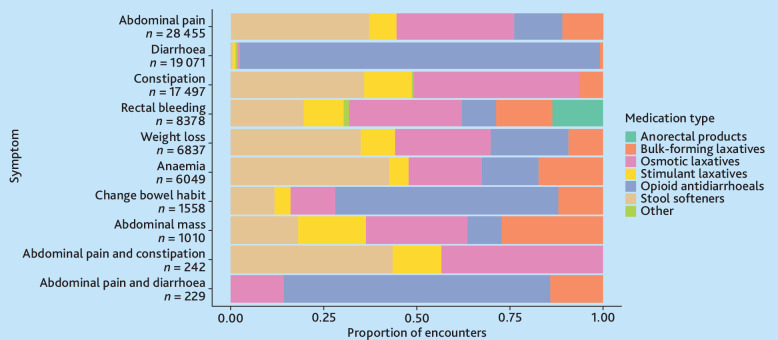
Variation of medication prescriptions ordered for colorectal cancer-related symptoms. Symptoms are listed in order of the most common colorectal cancer symptoms by reason for encounter.

Figure 4 presents the variation in medication prescribing patterns across the symptom groups. Osmotic laxatives (44%) and stool softeners (36%–44%) dominated constipation management. Opioid antidiarrhoeals were prescribed for 98% of diarrhoea and 60% of changes in bowel habits. Anorectal products were only prescribed for rectal bleeding (14%), while bulk-forming laxatives and stimulant laxatives were infrequently used across all symptoms.

### Characteristics associated with investigation or referral

The likelihood of investigation was associated with age, remoteness, socioeconomic status, test of treatment, and clinical complexity (all *P*<0.001), but not sex or smoking (Table 1). Investigation rates declined from 59% (aged 60–69 years) to 36% (aged ≥80 years). Rates increased with socioeconomic advantage (49% most disadvantaged versus 56% least disadvantaged). Test-of-treatment prescription reduced the likelihood of investigation (32% versus 54%). Clinical complexity strongly influenced investigation: 70% of patients with multiple symptoms and multiple encounters versus 48% with a single symptom and single encounter were investigated.

**Table 1.** Characteristics of patients who were and were not investigated (including pathology, imaging, and colonoscopy) or referred

Factor	Investigation (testing/referral)				*P*-value
	Yes		No		
	*n*	%	*n*	%	
**Total**	36 367	52	33 740	48	
**Sex**					
Female	14 288	52	13 440	48	0.14
Male	22 076	52	20 296	48	
**Age group, years**					
40–49	9662	52	8781	48	<0.001
50–59	8698	57	6612	43	
60–69	7562	59	5234	41	
70–79	5842	54	4995	46	
≥80	4603	36	8118	64	
**Remoteness**					
Major cities of Australia	21 612	52	19 907	48	<0.001
Inner regional Australia	12 779	52	11 682	48	
Outer regional and remote Australia	1913	48	2055	52	
**IRSD quintile**					
1 (most disadvantaged)	7682	49	8156	51	<0.001
2	7422	49	7636	51	
3	6781	51	6443	49	
4	6451	55	5232	45	
5 (least disadvantaged)	7945	56	6165	44	
**Medication for test of treatment**					
No	34 111	54	28 862	46	<0.001
Yes	2256	32	4878	68	
**Clinical complexity**					
Single encounter and single symptom	26 788	48	28 981	52	<0.001
Multiple encounters and single symptom	5969	66	3013	34	
Multiple encounters and multiple symptoms	3305	70	1441	30	
Single encounter and multiple symptoms	305	50	305	50	
**Smoking status**					
Non-smoker	18 741	54	16 248	46	0.29
Ever-smoker^b^	14 996	53	13 226	47	

^a^Missing observations were present in several categories but are not shown. ^b^Ever-smoker includes both current and ex-smokers. IRSD = Index of Relative Socioeconomic Disadvantage.

Multivariable regression revealed that patients aged ≥80 years had reduced odds of investigation (odds ratio [OR] 0.53, 95% confidence interval [CI] = 0.50 to 0.55), whereas those aged 50–79 years had higher odds than those aged 40–49 years (Table 2). The least disadvantaged patients had 45% higher odds of investigation than the most disadvantaged (OR 1.45, 95% CI = 1.38 to 1.53). This gradient varied by investigation type: patients who were disadvantaged received more primary care investigations, patients who were advantaged received more colonoscopy/specialist referrals (see Supplementary Table S1). Test of treatment reduced odds of investigation (OR 0.39, 95% CI = 0.37 to 0.42) (Table 2). Multiple symptoms and multiple encounters strongly predicted investigation versus single symptom and single encounter (OR 3.40, 95% CI = 3.16 to 3.65). Multilevel regression analysis showed substantial practice-level variation in investigation patterns.

**Table 2.** Results of multiple logistic regression model showing the odds ratio (OR) of factors associated with diagnostic testing or referral

Variable	OR	95% **CI**	*P*-value
**Age, years**			
40–49	Reference		
50–59	1.18	1.13 to 1.24	<0.001
60–69	1.32	1.20 to 1.38	<0.001
70–79	1.09	1.03 to 1.14	0.002
≥80 years	0.53	0.50 to 0.55	<0.001
**Remoteness**			
Major cities of Australia	Reference		
Inner regional Australia	1.15	1.10 to 1.19	<0.001
Outer regional and remote Australia	1.08	1.00 to 1.17	0.043
**IRSD quintile**			
1 (most disadvantaged)	Reference		
2	1.03	0.98 to 1.08	0.324
3	1.09	1.03 to 1.15	0.001
4	1.33	1.26 to 1.40	<0.001
5 (least disadvantage)	1.45	1.38 to 1.53	<0.001
**Medication for test of treatment**			
No	Reference		
Yes	0.39	0.37 to 0.42	<0.001
**Smoking status**			
Non-smoker	Reference		
Ever-smoker^a^	0.96	0.93 to 0.99	0.014
**Clinical complexity**			
Single encounter and single symptom	Reference		
Multiple encounters and single symptom	2.50	2.37 to 2.63	<0.001
Multiple encounters and multiple symptoms	3.40	3.16 to 3.65	<0.001
Single encounter and multiple symptoms	0.98	0.83 to 1.16	0.844

^a^Ever-smoker includes both current and ex-smokers. IRSD = Index of Relative Socioeconomic Disadvantage. OR = odds ratio.

The modelled OR covering the middle 95% of practices ranged from 0.34 to 2.94, representing an 8.6-fold variation between practice. This indicates that patients with similar characteristics could have very different chances of being investigated depending on which practice they attended. The intraclass correlation coefficient was 0.084, suggesting that 8.4% of the variation in investigation decisions was owing to practice-level factors rather than patient characteristics. After accounting for practice clustering, the remoteness effect was no longer significant, and socioeconomic differences became weaker, indicating these associations were partly explained by practice-level factors (see Supplementary Table S2).

### Colorectal cancer diagnosis

Of 70 107 patients, 378 (0.5%) were diagnosed with colorectal cancer within 12 months of symptom presentation. Anaemia had the highest positive predictive value (PPV) (1.8%, *n* = 74/4226), followed by rectal bleeding (1.3%, *n* = 86/6772). Abdominal pain, although common (*n* = 20 956), had a low PPV (0.3%). Median time to diagnosis was 46 days (interquartile range 16–116), shortest for abdominal mass (11 days) and change in bowel habit (23 days), longest for constipation (71 days) and diarrhoea (51 days). Combined abdominal pain and constipation showed a PPV of 1.1%, with a 41-day median diagnostic interval (see Supplementary Table S3).

## Discussion

### Summary

In this large primary care cohort of 70 107 patients with colorectal cancer-related symptoms, substantial variation in investigation and management practices was observed. Either no investigation or prescription occurred in 20%–50% of encounters, highest for constipation and diarrhoea. Tests of treatment predominated for diarrhoea and constipation, whereas blood and imaging tests were common for abdominal pain and abdominal mass. Colonoscopy/specialist referral was highest for rectal bleeding and change in bowel habit. Patients from disadvantaged areas received fewer investigations overall, with more primary care tests than specialist referrals. Patients aged ≥80 and <50 years were less likely to be investigated than those aged 50–79 years. Multiple encounters and symptoms strongly predicted investigation. The overall colorectal cancer diagnosis rate was only 0.5%, with even ‘red flag’ symptoms such as anaemia and rectal bleeding having PPV <2%, reflecting the diagnostic challenge faced by GPs in selecting patients for diagnostic colonoscopy.

### Strengths and limitations

To the authors’ knowledge, this is the largest study examining real-world investigation patterns for symptoms of colorectal cancer in primary care, using linked data from over 70 000 patients. The study addressed the challenge of variable symptom documentation in general practice by applying a transparent and systematic approach to classifying free-text 'reason for encounter' entries across multiple medical record systems, helping to ensure consistent symptom coding. Another key strength is the integration of general practice data with hospital admission and cancer registry datasets, enabling a comprehensive assessment of both primary care-led investigations and hospital-based procedures such as colonoscopy. Notably, colonoscopy data were derived from the VAED, allowing the authors to capture procedures conducted in both public and private hospitals.

Limitations include potential under-recording of symptoms documented in the ‘reason for encounter’ field rather than in free-text clinical notes. Prescribing data in the PATRON database do not always distinguish between new prescriptions and repeats, and may include regular medications unrelated to the presenting complaint. Finally, it was not possible to capture GP-level or system factors (such as workload, time constraints, and test access) that influence investigation decisions.

### Comparison with existing literature

Half of patients with constipation and diarrhoea received no investigation. These non-specific symptoms have a PPV <1% for colorectal cancer.^17^ ‘Tests of treatment’ are often used in diagnostic decisions relating to non‐acute undifferentiated presentations with robust safety-netting.^16,18–20^ UK evidence suggests direct referral without investigation can shorten diagnostic time, although applicability to Australia’s more flexible gatekeeping model is unclear.^21^ Low investigation rates for anaemia are concerning, given the association with colorectal cancer, although this may reflect variations in recording or follow-up practices.

The current cohort showed lower pathology testing (<40% across symptoms) compared with UK research reporting >60% blood test use.^22^ In one Australian study of patients with colorectal cancer, approximately one-quarter had a blood test requested in the 24 months before diagnosis, with a similar proportion showing abnormal results.^23^ Blood tests can provide important early diagnostic clues detectable up to 9 months before diagnosis.^23,24^

Relatively low levels of iFOBT testing was found (see Supplementary Figure S4). Although recommended in Australia for determining colonoscopy urgency, it is not consistently included across guidelines, contrasting with UK National Institute for Health and Care Excellence recommendations for triage testing.^13,25,26^

The substantial between-practice variation identified through multilevel modelling indicates that clinical decision making for investigating colorectal cancer symptoms is influenced not only by patient characteristics but also by practice-level factors. This 8.6-fold variation in investigation likelihood between practices at the 2.5th and 97.5th percentiles cannot be explained by patient characteristics alone. Such variation may reflect differences in clinician training and experience, practice culture, or access to diagnostic resources. Although some variation is appropriate and reflects clinical judgement tailored to individual circumstances, the extent of this variation suggests opportunities for more standardised, evidence-based approaches to investigating lower gastrointestinal symptoms.

Similar to UK findings, the current study identified lower investigation rates among patients aged ≥80 years and in socioeconomically disadvantaged quintiles.^27^ In contrast to the current findings, individuals living in rural areas of the UK were less likely to be investigated, but this may reflect different patterns of access to investigations and referral pathways between countries. Although limited access to diagnostic services in rural areas is often of concern, the current findings suggest that such barriers may not substantially influence GP testing behaviour in Australia.

Regarding colonoscopy referral, a UK study by Mendonca *et al* ^28^ found that much of the between-practice variation was explained by sociodemographic characteristics, with higher rates observed in practices serving older and more socioeconomically deprived populations.^28^ In the present cohort, colonoscopy use peaked at the age of 60–79 years, declining after 80 years. In addition, higher referral likelihood in patients who were less disadvantaged was observed. This finding is consistent with previous Australian data showing greater colonoscopy use in higher socioeconomic areas, associated with the ability to pay for faster access to endoscopy.^29^ The differences observed in relation to associations with rurality may be explained by the fact that the current study’s dataset relies on patient locations to define remoteness rather than the location where the procedure was performed. This suggests that rural patients, especially those from more advantaged areas, may travel outside their local area to undergo a colonoscopy at a hospital situated in a larger city centre.^29^

The current study’s findings revealed important age-related disparities in investigation rates. Patients aged 40–49 years had lower investigation rates despite rising colorectal cancer incidence in younger adults.^2^ This may reflect lower PPV of symptoms in younger patients, a lack of awareness about the rising incidence in the younger group, the commonality of lower gastrointestinal complaints, age-based referral guideline restrictions, and GPs’ concerns about over-referring.^30^ The recent NBCSP expansion to include those aged 45–49 years addressed the increased incidence in younger people,^3^ yet the data in the current study suggest that symptomatic younger patients may still face barriers to timely investigation. For patients aged ≥80 years, the notable decrease in investigation rates probably results from complex clinical choices that weigh competing health issues. A prior systematic review identified a link between increasing age and longer diagnostic delays or delays in investigating cancer symptoms, decisions that are often complicated by frailty, multiple health conditions, and cognitive impairment.^31^

From a symptom perspective, changes in bowel habit triggered colonoscopy rates nearly equivalent to rectal bleeding, indicating clinicians recognise this as a concerning pattern, distinct from diarrhoea or constipation alone.^6^ Patients with repeat presentations or multiple symptoms were more likely to be investigated, aligning with elevated colorectal cancer risk.^17^ This suggests GPs respond to accumulating risk through clinical reasoning processes such as pattern recognition, hypothetico-deductive reasoning, or gut feeling.^32^

### Implications for research and practice

This study highlights substantial variation in investigating colorectal cancer symptoms in Australian general practice. Although some variation reflects appropriate clinical judgement, inconsistent investigation, especially for non-specific symptoms, indicates potential missed diagnostic opportunities. The low anaemia investigation rate is concerning and warrants further study but raises broader concerns about managing abnormal test results in primary care.

Differences in investigation rates by age, rurality, and socioeconomic status point to potential inequities in diagnostic access and may contribute to the observed variations in colorectal cancer outcomes.^33^ These findings highlight the importance of implementing system-wide strategies to promote more consistent and evidence-based decision making. This includes developing structured diagnostic pathways, improving integration of decision support tools, and establishing mechanisms to enhance follow-up of abnormal results. Further research is necessary to explore the effects of current investigation practices on diagnostic intervals, stage at diagnosis, and cancer outcomes.

## Data

The de-identified data analysed in this study are not publicly available, and data used for this study can be shared subject to appropriate ethical approvals. All authors had full access to all of the data (including statistical reports and tables) related to the study.
